# Plasma Enhanced Chemical Vapour Deposition of Horizontally Aligned Carbon Nanotubes

**DOI:** 10.3390/ma6062262

**Published:** 2013-05-31

**Authors:** Matthew T. Cole, William I. Milne

**Affiliations:** 1 Department of Engineering, Cambridge University, 9 JJ Thomson Avenue, Cambridge CB3 0FA, UK; E-Mail: wim1@cam.ac.uk; 2 AIXTRON Ltd., Cambridge CB24 4FQ, UK; 3 Department of Information Display, Kyung Hee University, Seoul 130-701, Korea

**Keywords:** carbon nanotube, *in situ* horizontal alignment, plasma enhanced chemical vapor deposition, electric field

## Abstract

A plasma-enhanced chemical vapour deposition reactor has been developed to synthesis horizontally aligned carbon nanotubes. The width of the aligning sheath was modelled based on a collisionless, quasi-neutral, Child’s law ion sheath where these estimates were empirically validated by direct Langmuir probe measurements, thereby confirming the proposed reactors ability to extend the existing sheath fields by up to 7 mm. A 7 mbar growth atmosphere combined with a 25 W plasma permitted the concurrent growth and alignment of carbon nanotubes with electric fields of the order of 0.04 V μm^−1^ with linear packing densities of up to ~5 × 10^4^ cm^−1^. These results open up the potential for multi-directional *in situ* alignment of carbon nanotubes providing one viable route to the fabrication of many novel optoelectronic devices.

## 1. Introduction

Carbon nanotubes (CNTs), high aspect ratio graphitic nano-rods of concentrically nested graphene planes, have a wide range of novel properties that have been exploited to develop field emission electron sources [1,2], X-ray sources [3,4] and optical polarising media [5,6]. However, many of these applications require accurate horizontal alignment with high linear packing densities in order to engineer particular functionalities. CNTs can be aligned by post-synthesis alignment, where external forces such electric fields, such as by dielectrophoresis [7,8,9] orientate the nanotubes once dispersed onto a substrate, often by means of a liquid medium. Alignment through compression, rolling and shearing have proven viable [10] though precisely aligned linear arrays have proven challenging to fabricate. Nanomanipulation has been reported, though the processing involved is ultimately serial and requires extremely time-consuming electron microscopy monitoring [11]. Spin and drop casting of nanotube suspensions have shown moderate success [12,13]. Nevertheless, despite the CNTs in-plane alignment they have little or no linear directionality, which is to say that there is no controllable way to define the mean azimuth and that they are merely randomly orientated within the plane. Wei *et al.* [14] demonstrated the use of dielectrophoresis exploiting the weak nanotube dipole. An electric field, of the order of ~10 V µm^−1^ [15,16,17,18], is required to align solution dispersed nanotubes, whereas only ~1 V µm^−1^ [19,20,21] is necessary to achieve alignment during synthesis.

Various reported *in situ* alignment techniques have been reported to-date. Most are based on either graphoepitaxy [22,23,24,25], rational substrate design [26], magnetic field [27], gas flow [28,29,30,31] or electric field [16,19,21]. Graphoepitaxially is surface critical. This limits the broadness of the technique to a very limited, and rather costly, set of substrates, such as sapphire or quartz, though high degrees of alignment and uniformity have been evidenced [25,32]. Despite this the process involves time-consuming substrate preparatory processes. Rational substrate design, using elevated Si pillars, offers another possible approach [26]. Here the CNTs grow between elevated pillars. These micrometre pillars limit the practicality of the technique as well as restricting the maximum packing density. As a result, techniques based on magnetic fields, electric fields and gas flow have shown perhaps the most promise. They are rapid, parallel processes that offer simplicity and the ability to fabricate high density arrays.

Zhang *et al.* [20] and Ural *et al.* [19] reported similar alignment effects ascribed to electric fields of the order of 0.5–4.0 V µm^−1^ whereas Law *et al.* [16] determined that plasma induced self-biasing and the resultant surface charging effects on metallic electrodes were sufficient to stimulate alignment. It has been argued that the nanotubes highly anisotropic polarisability induced large dipole moments when they interact with the local electric field. This interaction produces large aligning torques which governs the growth orientation. Blaek *et al.* [33] estimated the electric field aligning force to be of the order of 10^−5^ nN, a force approximately four orders of magnitude greater than the weight of the catalyst particle, whilst Hertel *et al.* [34] showed that, for a 10 nm wide nanotube, a force >35 nN is necessary to overcome the van der Waals surface binding. Few comprehensive attempts have been made in the literature to explain the orientation mechanism involved. Chen *et al.* [35] proposed a so-called “kite mechanism”. Here the catalyst particle, located at the nanotube apex, is drawn in the direction of the prevailing electric field. When growth terminates the nanotubes relax and fall to the substrate where they become strongly bound. Huang *et al.* [28] presented a similar mechanism, whereas Yu *et al.* [36] postulated that charged species form bonds along the electric field direction and that the nanotubes can only grow if they align to the electric field. Tanemura *et al.* [37] suggested that the alignment effect may be a result of an excess of electrostatically attracted positive charge ions at the nanotubes tip. They concluded that the plasma, composed of positive ions as opposed to radicals or excited molecules, plays a decisive role in the alignment by reducing the lateral mechanical stress exerted on the nanotubes. In our previous work we demonstrated vertically aligned CNT synthesis using plasma enhanced chemical vapour deposition (PE-CVD) [38,39] where the alignment occurs concurrently with growth. Here we report on the underlying theoretical framework and consequent design of a PE-CVD reactor to facilitate *in situ* horizontal alignment of CNTs.

## 2. Results and Discussion

Catalyst activity was initially validated using samples grown by traditional thermal-CVD (T-CVD), at 700 °C for 900 s, and vertical PE-CVD, as per our earlier work [40]. No horizontal potential was applied in either case. CNTs grew to a nominal length of 3 µm with a diameter of ~75 nm (PE-CVD) and ~77 nm (T-CVD). To investigate the degree of interaction between the as-synthesised T-CVD CNTs and a local electric field, a DC electric field of ~6 V µm^−1^ was applied post-growth. The CNTs strongly interacted with the electric field and become increasingly laterally dispersed by up to 20 µm in some cases, though little alignment was evident. The similarly wide distribution of catalyst particles would also suggest that the catalyst also interacts strongly with the field and that tip growth is ultimately preferential, supporting the posits of Chen *et al.* [35]. T-CVD synthesis was then undertaken with a horizontal bias (~2 V/µm) applied during growth. Here, the pre-patterned catalyst dots showed little interaction with the field, most likely due to the dominance of thermal effects over the local electric field. Samples prepared with a Ni catalyst showed some horizontal tubes with lengths of up to 10 µm, though linear densities were low (~100 mm^−1^). We believe the variations in alignment to be the product of disparities in the electrode fabrication though alignment can certainly be attributed to the electric field given the extremely linear growth. Other than the low-linear densities, a major limitation to this local electric-field alignment is the requirement for microelectrodes patterned directly on the substrate. These structures interfere with post-growth processing and device fabrication. Substrate independent electrodes obviate these limitations. The electric field can then be applied across *entire* samples. To the best of the author’s knowledge, no one has hitherto demonstrated such global alignment horizontally. A new horizontal nanotube PE-CVD reactor was modelled, designed and fabricated.

**Figure 1:**
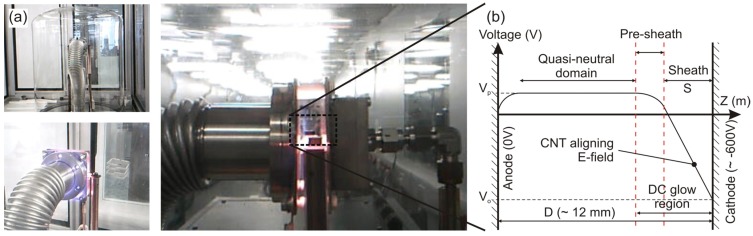
(**a**) Horizontal plasma enhanced chemical vapour deposition (PE-CVD) reactor; (**b**) Typical potential variation in a DC glow gas discharge plasma. (Typical process conditions: 50 W/700 V, 700 °C, 200:50 sccm, 3 mbar).

Figure 1 shows an optical micrograph of the reactor during a typical deposition. Samples were electrically isolated from the heated graphite stage using ceramic spacers to prevent parasitic charging of the underlying degenerately doped Si. The reactor consists of two 1.5 mm × 70 mm × 70 mm polished stainless steel electrodes. The cathode/gas inlet (right) is electrically grounded and the anode/gas outlet (left) is attached to a turbomolecular vacuum system that is electrically connected to a computer controlled variable high-voltage DC supply (0–1000 V). The electrode separation can be altered by adjusting the ceramic spacers as necessary. The sample is ohmically heated using a 0.13 mm × 10 mm × 100 mm graphite stage with a quartz, electrically isolating cover-sheet. The reactor was enclosed in an evacuated quartz bell jar.

It has been widely postulated that CNT alignment is mediated by anisotropic torque induction within a narrow plasma sheath [38]. This sheath exists in the narrow glow region, ~3 mm wide, adjacent to the biased cathode. For a collisionless, quasi-neutral, Child’s law ion sheath of electron temperature (*T_e_*) and glow potential (*V_p_*), the relation between the sheath width (*S*), charge carrier density (*n*) and cathode potential (*V_o_*) is given by [41,42];

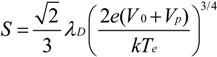
(1)
where ε_o_ is the permittivity of free space, *k* is Boltzmann’s constant and *e* is the electronic charge. Here the Debye length (*λ_D_*) is given by;

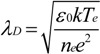
(2)
where *n_e_* is the electron density. In the presented reactor the Debye length is ~22 µm which is significantly smaller than the characteristic dimensions of the reactor (~1 mm). This validates our initial assumption of stable and largely collisionless plasma. Combining Equations (1) and (2), and assuming quasi-neutrality (*n* ~ *n_e_*), gives;

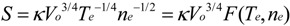
(3)
where *k* = (6.12 × 10^4^) m^5/2^V^−3/4^K^1/4^ and *F*(*T_e_*, *n_e_*) denotes the pressure and plasma power dependence of the electron temperature and the electron density. Note that the plasma potential *V_p_* << *V_0_*, where *V_p_* ~5 V and *V_0_* ≥ 500 V. Typical growth profiles (V_o_ ~ 600 V and *n_e_* ~ 10^17^ m^−3^) produce a 1.6 mm sheath for *T_e_* = 1.5 × 10^4^ K [41].

**Figure 2:**
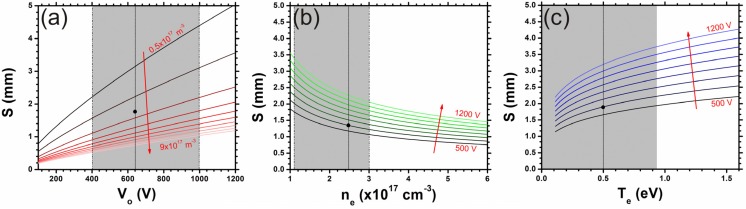
Theoretical sheath width dependence on (**a**) cathode bias (V_o_) and (**b**) electron density (*n_e_*) for an electron temperature (*T_e_*) ~10^4^ K (0.86 eV); (**c**) Variation in *T_e_*, with S for increasing V_o_. Points indicated denote typical process conditions for vertical PE-CVD nanotube synthesis over practical growth regimes (grey).

Equation (3) suggests that the sheath width is strongly dependent on the electron density and weakly dependent on the cathode bias. The derived dependencies are illustrated in Figure 2, where typical vertical nanotube synthesis bias (*V_0_* = 600 V) and electron density (*n_e_* = 8.2 × 10^16^ m^−3^) have been indicated (black dot) for clarity. Our estimates suggest that a ten-fold increase in sheath width is possible by tuning the cathode bias, electron temperature and electron density.

**Figure 3:**
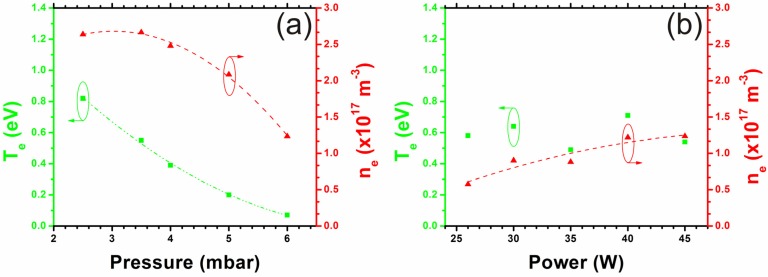
Pressure dependence variation in (**a**) electron density and electron temperature with reactor pressure. Functional fits as shown. (Typical plasma conditions: 45 W/550 V, 700 °C, 4:1 NH_3_:C_2_H_2_). Functional fits as indicated; (**b**) Power dependence variation in electron density and electron temperature with power. (Typical plasma conditions: 700 °C, 3.2 mbar, 200:50 sccm). Functional fits as indicated.

**Figure 4:**
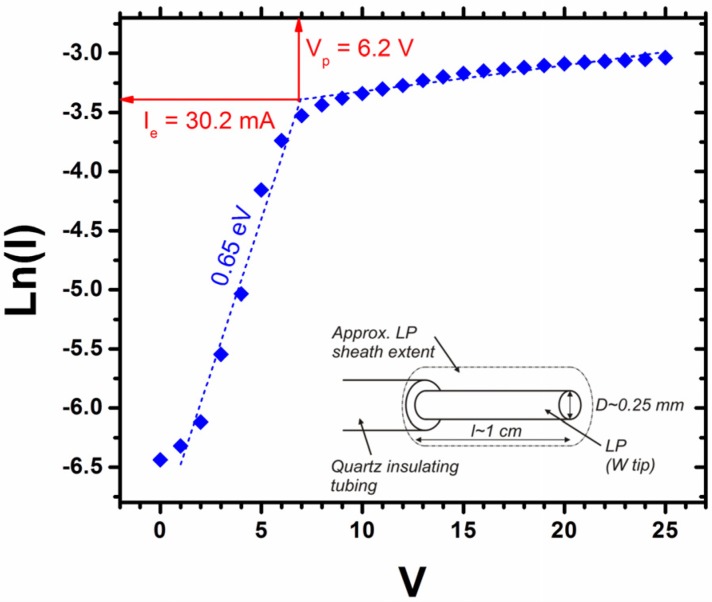
A typical Langmuir probe measurement (30 W, 3.3 mbar) showing the extracted probe current (I) at a given bias (V). Insert: Schematic depiction of the probe geometry.

We directly controlled the cathode bias and indirectly controlled the electron temperature and electron density by controlling the plasma power and reactor pressure. The measured variation in sheath width with plasma power and reactor pressure is shown in Figure 3. Here we have extrapolated the electron density and electron temperature as per our earlier discussion. Figure 4 shows a typical measured I–V characteristic (see Experimental Section). For equal electron and ion densities (*n_e_* ≈ *n*_+_ = *n*) the corresponding currents are;

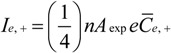
(4)
where the subscripts “e” and “+” refer to the electron and ion quantities, respectively, and *C* is the mean charge carrier velocity, given by;

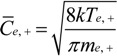
(5)
where the mass of the charge carriers (*m_e_*, *m*_+_) are known. The measured current is the sum of the electron and ion currents (|*I*|=|*I_e_*| = |*I*_+_|). However, *T_e_* >> *T*_+_ (∴ *C_e_* >> *C*_+_) , hence |*I*|≈|*I_e_*|. The electrons exhibit a Maxwellian energy distribution which modulates the current accordingly to give [43];

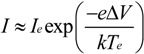
(6)
where Δ*V* (=*V_p_* − *V*) is the potential difference between the plasma and probe. The electron density can now be found from Equation (4). The parameters *V_p_*, *I_e_* and *T_e_* are empirically accessible through the Langmuir probe I–V data. We find that for typical process conditions (3.2 mbar/ 50 W/ 550 V), *n_e_* = 2.5 × 10^17^ m^−3^ and *T_e_* = 0.7 eV; values which show excellent agreement with those reported by Blaek *et al.* [33].

The electron density linearly increased with plasma power (Figure 4a), a similar trend to that reported by Desai *et al.* [44], and quadratically decays with pressure (Figure 4b). The electron temperature is largely independent of plasma power (Figure 4c) and exponentially decays, with a transient of 0.7 mbar^−1^, with increasing reactor pressure (Figure 4d). Sheath elongation is thusly viable by decreasing the plasma power. The electron density and temperature decrease with increasing reactor pressure, consistent with [44,45]. Increasing the reactor pressure tends to increase the sheath width. The measured trends conflict. To increase the sheath width the pressure must be increased (to decrease *n_e_*). However, the sheath width also increases at low pressures (to increase *T_e_*). Consequently, a more detailed functional dependence on *n_e_* and *T_e_* must be evaluated. Equations (7) and (8) show the functional fits, where *θ* denotes the plasma power, and where separation of variables has been assumed for *n_e_*(*P*, *θ*).

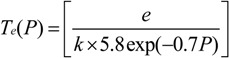
(7)

*n_e_*(*P*, *θ*) = *k*' *n_e_*(*P*)*n_e_*(*θ*) = (1.74×10^17^)(−0.16*P*^2^ +0.98*P* +1.19)(0.034*θ* − 0.23) (8)

Here, *k*' represents an averaging term which negates the scaling effects introduced by the assumed separation of variables. Equations (3), (7) and (8) can be combined to give a sheath width of the form *S* = *S*(*V_o_*, *P*, *θ*). Hence,

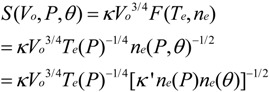
(9)
Functional fits to this theoretical framework suggest that a reactor pressure of 7 mbar and a plasma power of 25 W (~1000 V) are necessary to achieve a sheath width comparable to the substrate dimensions (~7 mm).

Catalyst samples were mounted with the gas flow parallel to the substrates surface. It is possible that this may enhance, or even account for any observed alignment, as per the findings of [29,30,31]. For turbulent flow the aligning effect of the electric field may be smeared out. The magnitude of the forces relating to the turbulent flow may very well dominate those associated with the electric field alignment, ~0.5 µN for nominally 45° misalignment [20]. The flow regime must therefore be considered. The Reynolds number is given by Re = *UL* / *υ* where *U* is the free-stream flow velocity, *υ* = *μ* / *ρ* is the kinematic viscosity (the ratio of the dynamic viscosity to density) and *L* is a characteristic dimension of the flow-perturbing feature. For an ideal gas a Reynolds number of ~100 is estimated, based on the data of Sun *et al.* [46] on the density and kinematic viscosity of what is a predominately an NH_3_ flow at 3.5 mbar (250 sccm)/700 °C, which indicates a predominately laminar flow (turbulent flow → Re > 2300). Thus, in principle, limited alignment degradation should occur as a result of the feedstock/passivation gases. Moreover, low gas flows (<300 sccm ~1.7 cm/s) were employed throughout to avoid turbulent interactions and to ensure the alignment was solely attributed to the electric field. Indeed, Xin *et al.* [31] showed, in a gas flow cell, free stream velocities of up to 9 cm/s were necessary for gas flow mediated alignment during T-CVD.

**Figure 5:**
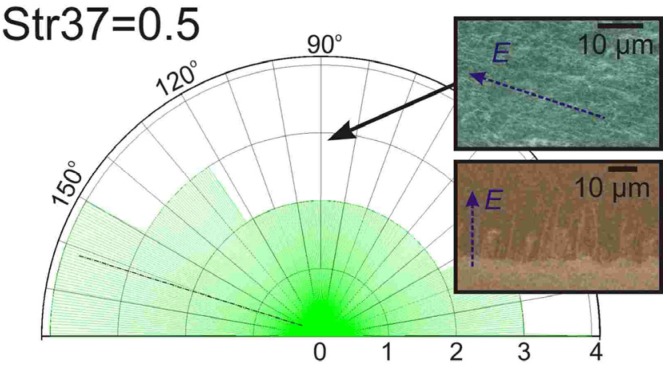
Typical SEM micrographs of an Al_2_O*_x_*/Fe sample grown in the horizontal PE-CVD reactor (435 V/15 W). E denotes the direction of the electric field. Quantified alignment giving a good mean alignment of Str37 = 0.5. (Scale bar: 10 μm)

The insets of Figure 5 shows typical SEM micrographs of the PE-CVD aligned CNTs (see Experimental Section). As in the local electric field case, surface-mediated growth passivation tended to occur in particular regions, inducing local unaligned zones. Nonetheless, samples showed a mean linear horizontal packing density of up to 5 × 10^4^ cm^−1^, consistent with the linear packing densities achieved by local electric field experiments reported elsewhere (0.2 × 10^4^ cm^−1 ^ – 9.2 × 10^4^ cm^−1^ [19,20,21]). The degree of alignment was evaluated using SPIP image analysis software, as previously detailed [10], and showed a mean Str37 value of 0.5 (Figure 5), where 0 denotes complete disorientation whilst 1.0 is for aligned arrays, which is comparable to the findings of [19,20,21].

**Figure 6:**
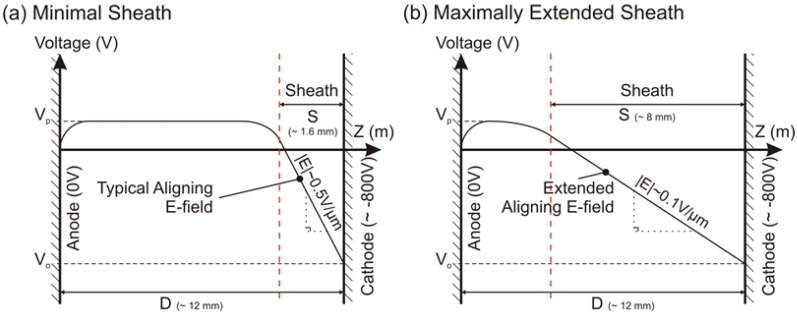
Illustrative examples of (**a**) typical and (**b**) optimal sheath width highlighting. The magnitude of the associated lateral electric field.

Consider the two extreme cases of sheath extension as illustrated in Figure 6. The global electric fields lie somewhere between 0.1 and 0.5 V/µm. The combined findings of [19,20,47,48] indicate that an electric field in the range of 0.1–2.0 V/µm is necessary to achieve alignment, values that are consistent with these extremities. Note, also, that Jang *et al.* [21] reported negligible improvements in the alignment “quality” for fields >2.0 V/µm, whilst Ural *et al.* [19] found an optimal field of 0.1 V/µm. The required fields are evidently accessible with the proposed reactor however high CNT areal density resulted in areas of misalignment due to strong randomising induced by inter-nanotube interactions (Figure 6a). Thus, a reduction in catalyst density would be favourable to increase the linear packing density and CNT length.

## 3. Experimental Section

Nanotubes were grown by thermal chemical vapour deposition using an etchant-to-feedstock gas ratio of 200:50 sccm (NH_3_:C_2_H_2_). W electrodes were patterned on Si/SiO_2_ (200 nm) substrates, separated by a 50 µm gap by standard photolithography. Ni catalyst strips and 80 nm catalyst dots (Al_2_O*_x_*/Fe–10/1 nm) were patterned between the electrodes using poly(methylmethacrylate) mediated electron beam lithography. W (25 nm) supported Fe (5 nm) samples were photolithographically defined and prepared by magnetron sputtering. Metallic supports were intentionally used to minimise the interaction between the growing nanotubes and the Si/SiO_2_ substrate. Catalyst samples were loaded into the reactor following an acetone and IPA degreasing step and nanotubes were grown at 700–800 °C.

Plasma characteristics were measured using a cylindrical Langmuir probe of length 10^−2^ m; radius, r = 0.25 × 10^−3^ m and exposed surface area, A_exp_ ~ 10^−5^ m^2^, as depicted in the insert of Figure 3. The probe was inserted into the plasma sheath under typical process conditions of 4:1 (C_2_H_2_:NH_3_) at 700 °C. The probes tip was 2–5 mm from the cathode centre in the glow discharge region. Following 300 s of plasma stabilisation the reactor pressure was reduced by either throttling the attached roughing pump or varying the input flow rates, followed by a second plasma stabilisation period of 300 s. The reactor pressure was varied from 2.5 to 6 mbar and the I–V characteristics were recorded using a Velleman DVM890. The probe bias was incrementally increased from 0 to 25 V using a Manson EP-613 source.

## 4. Conclusions

A new plasma-enhanced chemical vapour deposition reactor is reported offering a new route to synthesis horizontally aligned carbon nanotubes. The reactor does not require time consuming and costly substrate preparatory processes and can synthesis CNTs with an alignment factor of Str37 = 0.5. A model based on a collisionless, quasi-neutral ion sheath is presented and has been validated by direct Langmuir probe measurements confirming the proposed reactors ability to extend the existing sheath fields by up to 7 mm using a 7 mbar, C_2_H_2_:NH_3_ growth atmosphere with a plasma power of 25 W. The presented reactor permits the concurrent growth and alignment of carbon nanotubes with electric fields of the order of 0.04 V μm^−1^ with linear packing densities of up to ~5 × 10^4^ cm^−1^. These results open up the potential for multi-directional *in situ* alignment of carbon nanotubes providing one viable route to the fabrication of many novel optoelectronic devices.
